# Self-Reported Side Effects following Mass Administration of Azithromycin to Eliminate Trachoma in Amhara, Ethiopia: Results from a Region-Wide Population-Based Survey

**DOI:** 10.4269/ajtmh.18-0781

**Published:** 2019-01-21

**Authors:** Tigist Astale, Eshetu Sata, Mulat Zerihun, Andrew W. Nute, Aisha E. P. Stewart, Melsew Chanyalew, Berhanu Melak, Zebene Ayele, Demelash Gessese, Gedefaw Ayenew, Bizuayehu Gashaw, Zerihun Tadesse, Elizabeth Kelly Callahan, Scott D. Nash

**Affiliations:** 1 The Carter Center Ethiopia, Addis Ababa, Ethiopia;; 2 The Carter Center Atlanta, Atlanta, Georgia;; 3 Amhara National Regional Health Bureau, Bahir Dar, Ethiopia

## Abstract

A region-wide population-based post–mass drug administration (MDA) coverage survey was conducted 3 weeks following the 2016 trachoma MDA in Amhara, Ethiopia. The prevalence of self-reported side effects was assessed among those who self-reported receiving azithromycin. A total of 16,773 individuals from 5,129 households reported taking azithromycin during the 2016 MDA in Amhara. The regional prevalence of any self-reported side effect was 9.6% (95% CI: 8.3–11.2%) and ranged from 3.9% to 12.4% among the 10 zones. The most common reported side effects were abdominal pain (53.1%), nausea (21.7%), vomiting (12.8%), and diarrhea (12.5%). Side-effect prevalence among female members was higher than in male members (11.6% versus 7.6%; *P* < 0.001) and increased with age. After an average of 8 years of annual MDA, the prevalence of self-reported side effects was less than 10% in this population.

Mass drug administration (MDA) with azithromycin is one of the components of the WHO-endorsed surgery, antibiotics, facial cleanliness, and environmental improvement (SAFE) strategy to eliminate trachoma as a public health problem.^1^ Under this strategy, all community members aged ≥ 6 months within trachoma-endemic administrative districts are eligible for MDA.^1^ The Trachoma Control Program in Amhara, Ethiopia, was launched in 2003, and the full SAFE strategy, including annual MDA, was scaled up to the entire region by 2010.^2^ As of 2016, districts in Amhara have received between six and 11 rounds of MDA depending on when the SAFE scale-up occurred (approximately 131 million doses of azithromycin).

Although high coverage is important for the success of an MDA program, key structural and behavioral barriers often exist which can hamper the effectiveness of this intervention.^3,4^ A fear of side effects has been identified by community members as a reason for not participating in neglected tropical disease MDAs, including trachoma.^4–8^ Despite the excellent safety profile of azithromycin, a clearer understanding of the magnitude of reported side effects of azithromycin would be useful to guide trachoma control programs in designing specific strategies aimed at overcoming this barrier to high coverage.^9^ This survey estimated the prevalence of self-reported side effects from a population-based sample following a trachoma MDA in Amhara, a region with a mature trachoma control program.

The Trachoma Control Program in Amhara follows the WHO-recommended MDA schedule and dosing. Each year in eligible districts, all consenting community members were offered either a single oral dose of azithromycin (1 g for adults and 20 mg/kg for children approximated by height) or a course of tetracycline eye ointment (TEO) according to national guidelines. Tetracycline eye ointment is provided to self-reporting pregnant women in their first trimester, infants aged < 6 months, persons with serious illness, those who previously experienced adverse drug reaction to azithromycin, and to participants who decline oral azithromycin.

In 2016, a region-wide population-based coverage survey was conducted approximately 3 weeks following the trachoma MDA in Amhara. As detailed previously, a multistage cluster randomized sampling design was implemented to allow for zonal coverage estimates for each of the 10 zones in Amhara.^4^ A total of 32 clusters (villages) were selected in each zone. Informed consent was obtained from the head of participating households or from another adult household member. Each household member was presented with a medication sample and asked about taking the drug. The distributions of trachoma medications were not integrated with other medications during the MDA. A secondary outcome of this survey was to estimate the prevalence of self-reported side effects among those who reported taking azithromycin. Parents or caretakers responded for children aged < 10 years or for those who could not respond for themselves.

Zonal prevalences of self-reported side effects were estimated using survey weights that accounted for selection probability. CIs were calculated using Taylor linearization, and survey procedures in Stata, version 13.1 (STATA Corporation, College Station, TX), were used to account for clustering. To assess clustering of side effects, we calculated the intraclass correlation coefficient (ICC) with a random-effects logistic regression model without the inclusion of independent variables.

Among the 20,932 individuals from 5,184 households who were present at the time of the region-wide survey, 16,773 (76.8%, 95% CI: 69.3–82.9%) reported taking the MDA medications (zonal prevalence range 68–90%).^4^ Among those who reported taking azithromycin, the regional prevalence of any self-reported side effects among all age groups was 9.6% (95% CI: 8.3–11.2%) and 4.7% (95% CI: 3.6–6.2%) among children aged 1–9 years. A greater amount of clustering was observed at the household level (ICC = 0.32, 95% CI: 0.28, 0.37) than at the village level (ICC = 0.16, 95% CI: 0.13, 0.20). Zonal-level side effects ranged from 3.9% (95% CI: 2.5–6.2%) in Oromia to 12.4% (95% CI: 9.4–16.1%) in South Wollo (Table 1).

**Table 1:** Zonal-level self-reported side effects of azithromycin, Amhara, Ethiopia, 2016

Zone	Total, *N*	Whole population (95% CI)*	Total, *N* (1–9 years)	Children 1–9 years (95% CI)*
Awi	1,481	7.3% (5.2–10.3%)	422	4.8% (3.0–7.7%)
East Gojjam	1,598	7.0% (4.1–11.6%)	381	3.1% (1.1–8.1%)
North Gondar	1,562	10.0% (7.3–13.4%)	465	6.0% (3.7–9.7%)
North Shoa	1,694	9.9% (6.9–13.9%)	387	3.7% (1.6–8.2%)
North Wollo	1,682	12.3% (9.0–16.5%)	429	7.5% (4.8–11.6%)
Oromia	1,928	3.9% (2.5–6.2%)	657	2.1% (1.1–4.2%)
South Gondar	1,601	10.7% (7.0–16.1%)	374	5.0% (2.8–8.8%)
South Wollo	1,618	12.4% (9.4–16.1%)	374	3.6% (2.1–6.3%)
West Gojjam	1,461	4.8% (3.7–6.1%)	374	1.7% (0.7–4.2%)
Waghimra	1,749	11.0% (7.9–15.2%)	622	5.0% (2.9–8.3%)

* Weighted zonal estimate. Multilevel survey design accounted for in analysis.

Side-effect prevalence among female members was higher than among male members (11.6% versus 7.6%; *P* < 0.001). This difference was age dependent whereby there was little difference among children aged 1–9 years (4.4% in male members and 5.0% in female members), whereas the difference in prevalence in the child-bearing age groups was clearer as CIs did not overlap, 14.7% (95% CI: 11.4–18.6%) in female members versus 6.0% (95% CI: 4.1–8.7%) in male members for the age group 20–29 years; and 14.8% (95% CI: 11.8–18.4%) in female members versus 8.2% (95% CI: 5.8–11.7%) in male members for the age group 30–39 years (Figure 1). Reported side effects increased with age: 2.7% (95% CI: 0.9–8.0%) in those younger than 1 year, to 14.7% (95% CI: 10.8–19.7%) in those aged > 49 years. Reported side effects in those who took TEO was 0.8% (95% CI: 0.3–2.2%).

**Figure 1.**
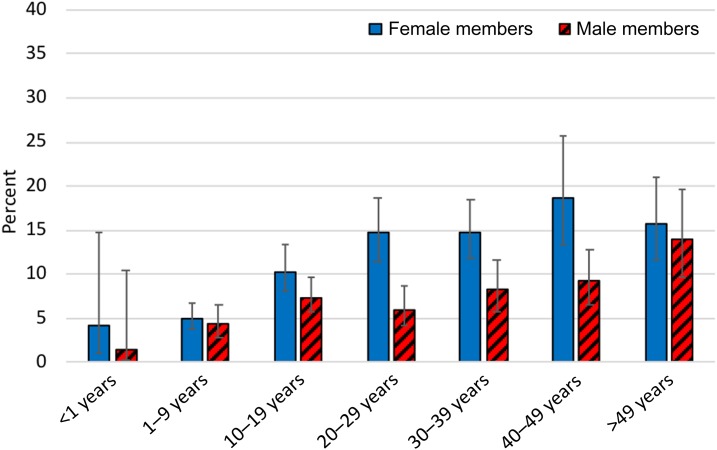
Self-reported side effects of azithromycin by gender and age group, Amhara, Ethiopia, 2016. This figure appears in color at www.ajtmh.org.

Among those reporting a side effect, a majority, 76.0% (95% CI: 69.5–81.5%), listed only one side effect and 24.0% (95% CI: 18.5–30.5%) listed two or more side effects. The most commonly reported side effects were abdominal pain (53.1%), nausea (21.7%), vomiting (12.8%), and diarrhea (12.5%) (Figure 2). In the five zones of the East Amhara subregion, people who reported side effects not included in the survey as possible responses were asked to further explain their “other” response. Of the 115 participants who reported “other,” the three most common responses were headaches (29.7%), chest pains (17.2%), and vertigo (17.2%).

**Figure 2.**
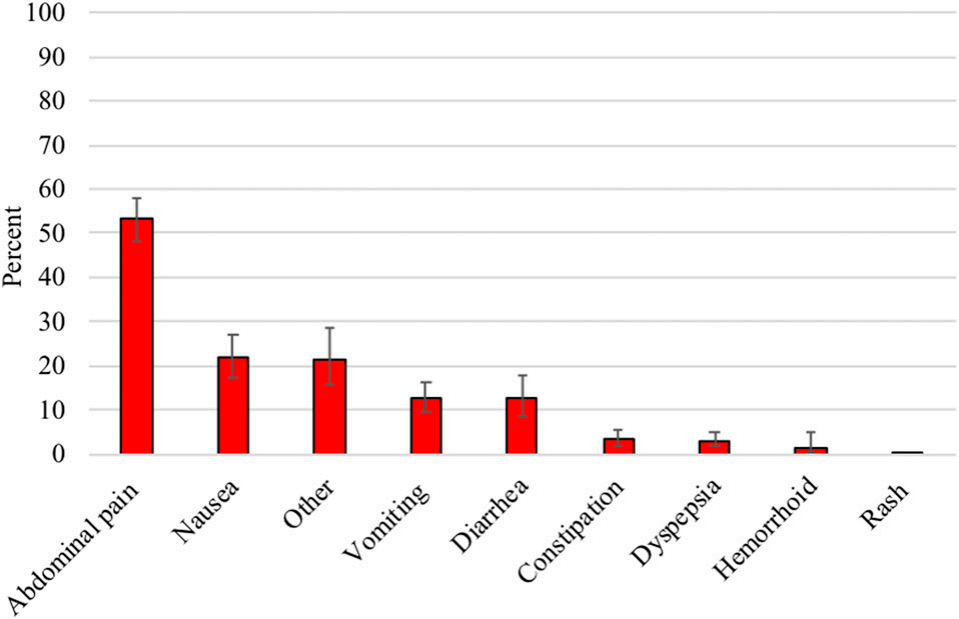
Most commonly reported side effects of azithromycin, Amhara, Ethiopia, 2016. This figure appears in color at www.ajtmh.org.

In this region-wide population-based survey of nearly 17,000 individuals, the prevalence of self-reported side effects was 9.6% among all age groups and 4.7% among children aged 1–9 years demonstrating that after numerous rounds of MDA in Amhara, reported medication side effects were low compared with a previous report from the same region.^10^ These data provide further evidence that azithromycin remains a safe, well-accepted medication for use in trachoma control programs, particularly among the key demographic of young children.

Previous reports of side effects of azithromycin for different diseases, including trachoma, varied across studies ranging from 0% to 41.0%.^10–18^ Direct comparison with these studies is difficult because of the differences in study settings or target populations. In one study conducted in 12 communities in a trachoma-endemic district of Amhara, adverse event surveillance indicated a prevalence of 7.0% in children aged 1–9 years and 18.7% in persons aged ≥ 10 years.^10^ Our survey was conducted to better understand the side effects among a sample relevant for large trachoma control programs. Several zones had a prevalence of side effects > 10%, and thus increased follow-up and health education may be warranted before a fear of side effects becomes a barrier to high coverage. The most commonly reported side effects within our survey were similar to those reported in different trachoma-specific settings.^10,14^ Although these side effects are not ideal, it has been noted that some beneficiaries take these side effects as a sign that the medications are working.^14^

Within Amhara, the prevalence of self-reported MDA coverage was high in children aged 1–9 years,^4^ whereas prevalence of side effects in the same age group was lower than that in older age groups. These findings are promising as these children are the primary focus of the Trachoma Control Program. The fact that these data come from communities which have received numerous rounds of MDA also suggests that despite the longevity of the program, the medication is accepted and tolerated. As noted previously, lower side effects among children may be because of reporting bias if survey respondents, normally parents or heads of household, underreported or underestimated symptoms in young children.^10^ A recent report, however, has demonstrated that beyond its effect on reducing chlamydial infection, MDA with azithromycin has been shown to reduce childhood mortality, particularly in the youngest children.^19^ The minor side effects noted among this population may be acceptable given these positive benefits of azithromycin.

The overall prevalence of self-reported side effects was significantly higher among female members than male members in Amhara. This relationship was suggestive, but not statistically significant in an earlier report from the region.^10^ The higher rates of side effects reported by women in this survey may be related to the reporting of health symptoms associated with pregnancy. Our data suggested that the difference between female members and male members was age dependent, with the greatest differences observed in the child-bearing age groups, particularly those aged 20–29 years and 30–39 years. Women beyond the first trimester of their pregnancies may have taken azithromycin during this campaign and may have reported pregnancy-associated health symptoms at the time of this survey. Unfortunately, it was not possible to identify pregnant participants within this survey.

This study was conducted in a random sample of households representing all age groups following community-wide MDA, thus providing important data for the Amhara Trachoma Control Program. Limitations of this survey include the fact that it was hard to verify self-reported side effects, especially in young children where response from caretakers was relied on. Furthermore, general illness was not assessed in MDA non-participants, which would have helped contextualize the magnitude of side effects among participants.

In summary, a < 10% prevalence of side effects reported from communities which have received numerous MDAs in Amhara is encouraging because trachoma MDA programs may take longer to reach their goals than the guidelines suggest and because MDAs with azithromycin may at some point be considered a childhood mortality prevention intervention.^2,19^
